# Age and Sex Influence the Neuro-inflammatory Response to a Peripheral Acute LPS Challenge

**DOI:** 10.3389/fnagi.2019.00299

**Published:** 2019-11-05

**Authors:** Valentina Murtaj, Sara Belloli, Giuseppe Di Grigoli, Maria Pannese, Elisa Ballarini, Virginia Rodriguez-Menendez, Paola Marmiroli, Andrea Cappelli, Valeria Masiello, Cristina Monterisi, Giuseppe Bellelli, Paola Panina-Bordignon, Rosa Maria Moresco

**Affiliations:** ^1^PhD Program in Neuroscience, School of Medicine and Surgery, University of Milano-Bicocca, Monza, Italy; ^2^PET and Nuclear Medicine Unit, San Raffaele Scientific Institute, Milan, Italy; ^3^Institute of Molecular Bioimaging and Physiology of National Reasearch Council, Segrate, Italy; ^4^Neuroimmunology Unit, Division of Neuroscience, IRCCS San Raffaele Scientific Institute, Milan, Italy; ^5^Milan Center for Neuroscience, School of Medicine and Surgery, University of Milano-Bicocca, Monza, Italy; ^6^Department of Medicine and Surgery, Tecnomed Foundation, University of Milano-Bicocca, Monza, Italy; ^7^Department of Biotechnology, Chemistry and Pharmacy, University of Siena, Siena, Italy; ^8^Acute Geriatric Unit, School of Medicine and Surgery, San Gerardo Hospital, University of Milano-Bicocca, Monza, Italy; ^9^School of Medicine and Surgery, San Raffaele Vita-Salute University, Milan, Italy

**Keywords:** aging, neuroinflammation, microglia, astrocytes, 18 kDa translocator protein, triggering receptor expressed on myeloid cells 2

## Abstract

Aging is associated with an exaggerated response to peripheral inflammatory challenges together with behavioral and cognitive deficits. Studies considering both age and sex remain limited, despite sex dimorphism of astrocytes and microglial cells is largely recognized. To fill this knowledge gap, we investigated the effect of a single intraperitoneal lipopolysaccharide (LPS) administration in adult and aged mice. We assessed the expression of different inflammatory mediators, and the microglial response through binding of [^18^F]-VC701 tracer to translocator protein (TSPO) receptors in the male and female brain. Aged female brain showed a higher pro-inflammatory response to LPS compared to adult female and to aged male, as revealed by *ex vivo* binding to TSPO receptors and pro-inflammatory mediator transcript levels. The highest astroglial reaction was observed in the brain of aged females. Differently to the other groups of animals, in aged males LPS challenge did not affect transcription of triggering receptor expressed on myeloid cells 2 (TREM2). In conclusion, our study shows that in the mouse’s brain the neuro-inflammatory response to an acute peripheral insult is sex- and age-dependent. Moreover, our results might set the basis for further studies aimed at identifying sex-related targets involved in the modulation of the aberrant neuro-inflammatory response that characterizes aging. This knowledge could be relevant for the treatment of conditions such as delirium and dementia.

## Introduction

The activation of the peripheral immune system is reflected by a pro-inflammatory milieu in the central nervous system (CNS; Norden and Godbout, 2013). The mechanisms of neuroinflammation are the subject of an intense pre-clinical research effort. However, most studies focus only on male animals, as preferred sex. A recent systematic review showed that only 3 out of 51 publications included both male and female mice or rats, and only a few of them focused on aged animals (Hoogland et al., 2015). This issue is relevant since ample evidence support sex-related differences in age-dependent neurodegenerative disorders (Bouman et al., 2005; Marriott and Huet-Hudson, 2006; Azad et al., 2007; Ycaza Herrera and Mather, 2015; Vom Steeg et al., 2016). Precise regulation of the immune responses maintains brain tissue homeostasis, while a chronic inflammatory state may influence the loss of neuronal function and plasticity (Besedovsky and Rey, 2008). In the presence of immune senescence, systemic inflammation represents a major precipitating factor for cognitive disorders (Perry, 2004; Costantini et al., 2018).

A neuro-inflammatory response induced by peripheral challenge with lipopolysaccharide (LPS) has been recently demonstrated in the brain of adult healthy volunteers using Positron Emission Tomography (PET) and a radio ligand for *in vivo* imaging of brain inflammation (Sandiego et al., 2015). However, most evidence on molecular and pathological CNS effects induced by peripheral inflammatory challenges, particularly during aging, derive from pre-clinical studies on rodents (Cunningham and Maclullich, 2013). As shown in the literature, aging is associated with an exaggerated response to peripheral inflammatory challenges together with behavioral and cognitive deficits (Hoogland et al., 2015; Schreuder et al., 2017). Indeed, in neurodegenerative disorders as well as in normal aging, microglia cells lose their supportive role in neuroplasticity and undertake a primed over-reactive phenotype promoting cognitive decline and synaptic dysfunction (Godbout and Johnson, 2006; Maclullich et al., 2008; Teeling and Perry, 2009). A recent gene expression profiling of microglia showed that aging is associated with over-expression of immune-related genes with an intermediate signature between acute and primed microglial genes (Holtman et al., 2015).

The association between genes regulating monocytes or microglial response with neurodegenerative disorders also supports the major role that neuroinflammation exerts in cognitive dysfunction. An example of this is the Triggering Receptor Expressed on Myeloid (TREM), a key component of innate and adaptive immunity, which is expressed by a variety of innate cells of the myeloid lineage including neutrophils, monocytes, osteoclasts, macrophages, dendritic cells and microglia. In particular, TREM2 has been shown to bind to poly-anionic ligands such as bacterial LPS and phospholipids (Wang et al., 2015). Upon ligand binding, TREM2 signals intracellularly through the adaptor protein DAP12, eventually regulating different cellular functions like phagocytosis, cytokine production, proliferation and survival (Thankam et al., 2016). Genetic studies have identified TREM2 variants that are associated with an increased risk of Alzheimer’s disease (AD; Zheng et al., 2018). Another protein of potential interest is the TREM cells Like 2 (TREML2 also named TLT2). TREML2 is upregulated on B cells, neutrophils and macrophages during inflammation, and recent data suggest a potential modulatory role in pro-inflammatory responses (Thomas et al., 2016). Indeed, a missense variant of TREML2 (rs3747742) has been associated with a reduced susceptibility to develop AD (Benitez et al., 2014; Bhattacharjee et al., 2014; Zhao and Lukiw, 2015). Females have a higher prevalence of AD compared to males, thus sex is included among the risk factors for dementia (McCarthy et al., 2012). Using *in vivo* imaging, Mosconi et al. (2017) demonstrated the presence of AD endo-phenotypes in the brain of asymptomatic peri-menopausal or menopausal women when compared to age-matched men.

Sex dimorphism of astrocytes and microglial cells is largely recognized and has been recently demonstrated by Villa et al. (2018). Adult female microglial cells carry a neuroprotective phenotype even when transplanted into male brain (Amateau and McCarthy, 2002; Hanamsagar et al., 2017; Villa et al., 2018). Interestingly, this protective phenotype seems to be in contrast with what has been observed in aged subjects, as suggested by a whole genome profile showing that old female brains exhibit higher transcription of genes of the complement system when compared to old males. The different neuro-inflammatory signatures may explain the sex-specific susceptibility to cognitive disorders (Mangold et al., 2017).

Translocator protein (TSPO) has been shown to be a reliable biomarker of microglia activation in a large number of studies of neuro-inflammation by pre-clinical imaging (Liu et al., 2014). During brain injury or inflammatory insults, TSPO is overexpressed in activated microglia cells. For this reason, TSPO ligands for PET imaging have been extensively used for the *in vivo* monitoring of microglia activation/macrophage infiltration in different neuropsychiatric conditions as well as in neurodegenerative and neuro-inflammatory animal models (Liu et al., 2014).

Currently, not only is there a lack of studies on the inflammatory responses in males and females but also studies considering both sex and age remain limited. For this reason, the main goal of this study was to test whether sex and age influence the early brain response to an acute peripheral inflammatory challenge. This experimental setting could reproduce the delirium syndrome, a transient and serious neurocognitive disorder characterized by an acute onset and fluctuating course (Inouye et al., 2014) To this aim, we investigated the effect of a single intraperitoneal LPS administration in male and female, adult and aged mice.

## Materials and Methods

### Animals and LPS Challenge

**Figure 1:**
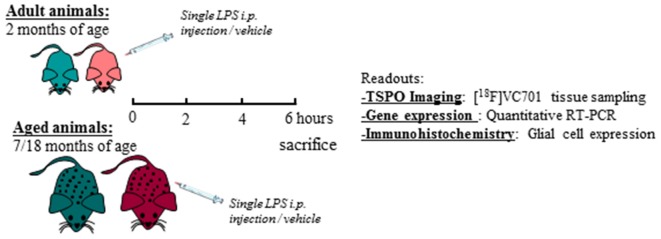
Schematic diagram of the experimental design and readouts.

Adult (2 months) and aged (17–18 months) non-breeding male and female C57Bl/6J mice were purchased from Charles River. Animals were maintained and handled in compliance with our institutional guidelines for the care and use of experimental animals (IACUC) and the national law for animals used in research (Prot. N. SK552/2012 D.lsg. 116/1992 and N. 722/2016-PR D.lsg. 26/2016). Mice were housed in the San Raffaele Hospital animal facility, maintained in 12/12 h light/dark cycle with access *ad libitum* to food and water. Following 1 week of acclimation, one group of 2 months old mice (adult) were processed according to the experimental tasks described below, whereas another group of mice was maintained until reaching the age of 17–18 months (aged) and were further processed. A total number of 76 mice were included in the study. For each experimental task, adult and aged mice of both sexes were randomly assigned to LPS or vehicle (saline) treatment as reported in Table 1 and in the diagram below (Figure 1). Animals were intraperitoneally injected with 0.63 mg/kg of LPS (Lipopolysaccharides of *Escherichia coli*, serotype 055:B5; Sigma-Aldrich) freshly dissolved in sterile saline prior to injection, or vehicle (2 mg/5 ml saline), and neuro-inflammatory responses were evaluated 6 h later (Biesmans et al., 2013).

**Table 1:** Age, body weight, sample size and treatment dosage of each experimental group.

Experimental group	Age	Body weight (g)	[^18^F]-VC701 binding (*n*)	RT-PCR (*n*)	IHC (*n*)	Dosage (LPS/vehicle)
Adult males + LPS	2 months	26.9 ± 2.7	8	5/8	3	0.63 mg/kg
Adult males vehicle	2 months	26.1 ± 1.6	8	5/8	3	50 μl saline
Adult females + LPS	2 months	18.3 ± 3.6	8	4/8	3	0.63 mg/kg
Adult females vehicle	2 months	19.6 ± 1.1	8	5/8	3	30 μl saline
Aged males + LPS	17/18 months	35.5 ± 2.5	8	5/8	3	0.63 mg/kg
Aged male vehicle	17/18 months	36.4 ± 3.3	8	5/8	3	50 μl saline
Aged females + LPS	17/18 months	31.1 ± 4.2	8	5/8	3	0.63 mg/kg
Aged females vehicle	17/18 months	34.2 ± 6.6	8	5/8	3	50 μl saline

### *Ex vivo* Binding of [^18^F]-VC701 to TSPO Protein

There were eight mice per group and treatment was dedicated to *ex vivo* imaging studies. Microglia activation was evaluated using the [^18^F]-labeled VC701, a TSPO radio ligand developed in our facility (Di Grigoli et al., 2015; Belloli et al., 2018). This radiopharmaceutical binds to 18 kDa TSPO. In this study, we used the novel TSPO tracer [^18^F]-VC701 that allows to obtain adequate signal to noise ratios for the measurements of TSPO binding in mouse brain (Belloli et al., 2018). Binding of [^18^F]-VC701 was evaluated *ex vivo* at 6 h after the intraperitoneal administration of LPS or saline. Animals were injected with 4.55 ± 1.85 MBq (specific activity: 3.17 ± 1.4 Ci/μMole) of [^18^F]-VC701 through the tail vein and sacrificed under light anesthesia 120 min later. Blood samples were collected and counted in a gamma counter (LKB Compugamma CS1282, Wallac). Brains were rapidly removed and divided into two hemispheres. Cortex, hippocampus and cerebellum were collected and placed in pre-weighed tubes. Radioactivity concentration was counted while the tissue used for RT-PCR was stored at −80°C for transcript measurement. After counting, tissue and blood radioactivity concentration were corrected for the half-life of [^18^F] (108.9 min) and expressed as percentage of the injected dose per gram of tissue (%I.D./g). To avoid a potential confounding effect due to differences in blood circulating levels, binding data were also normalized to blood uptake value.

### RNA Extraction and RT-qPCR Analysis

RT-PCR analysis was performed on five out of the eight animals/group that was used for the [^18^F]-VC701 experiments. One mouse in the adult female LPS-treated group was excluded due to insufficient mRNA availability. The brain areas (cortex, striatum, hippocampus and cerebellum) were dissected, homogenized in the supplied homogenization buffer and stored at −80°C. Total RNA was extracted with the Promega Maxwell^®^ 16 LEV simplyRNA kit using the Maxwell^®^ 16 Instrument (Promega). cDNA synthesis was performed using 250 ng of total RNA with the ThermoScript RT-PCR System (Invitrogen) and Random Primers (Promega) in a final volume of 20 μl according to the manufacturer’s instructions. The cDNA from each sample was used to perform RT-qPCR using LightCycler 480 SYBR Green I Master Mix (Roche) on the LightCycler 480 Instrument (Roche). β-actin was used as housekeeping gene for sample normalization. All analyses were performed in triplicate. The 2^−ΔΔCT^ method was used to calculate the relative changes in gene expression in LPS-treated relative to vehicle-treated mice. The two-way ANOVA following by Tukey *post hoc* test was used to evaluate statistical significance between LPS-treated groups while parametric unpaired Student’s *t*-test were used to compare LPS-treated to control group. Genes and relative primer sets used are listed in Table 2.

**Table 2:** Genes and relative primer list used in gene expression analysis.

Name	Primer sequence (5′-3′)
*β-actin F*	5′-gactcctatgtgggtgacgagg-3′
*β-actin R*	5′ catggctggggtgttgaaggtc-3′
*Trem2 F*	5′-gcacctccaggaatcaagag-3′
*Trem2 R*	5′-gggtccagtgaggatctgaa-3′
*Trem2L F*	5′-tggtggtggtgttgacatttcttcc-3′
*Trem2L R*	5′atccagggtttagcatagttgctgc-3′
*Tspo F*	5′-tcagcggctaccaacct-3′
*Tspo R*	5′-caggattcaggcatggtgat-3′
*Il-1β F*	5′-gcccatcctctgtgactcat-3′
*Il-1β R*	5′-aggccacaggtattttgtcg-3′
*Tnf-α F*	5′-cctgtagcccacgtcgtag-3′
*Tnf-α R*	5′-gggagtagacaaggtacaaccc-3′
*Il4 F*	5′-tcaacccccagctagttgtc-3′
*Il4 R*	5′-tgttcttcgttgctgtgagg-3′
*Arg1 F*	5′-ttgggtggatgctcacactg-3′
*Arg1 R*	5′-gtacacgatgtctttggcaga-3′
*IL-6 F*	5′-agttgccttcttgggactga-3′
*IL-6 R*	5′-tccacgatttcccagagaac-3′

### Immunohistochemistry (IHC)

Immunohistochemistry (IHC) and histopathologic analysis were performed in distinct adult and aged female mice (*n* = 3 per group and treatment). Mice were sacrificed 6 h after LPS or vehicle challenge and perfused with PBS followed by 4% paraformaldehyde (PFA). Brains were removed, post-fixed in PFA 4% for 24 h, cryo-protected overnight in 20% sucrose and 10 μm sections were cut on a cryostat for histological analysis in bright-field microscopy, slices were stained using standard protocols for hematoxylin and eosin (using Mayer’s Hematoxylin, BioOptica #05-06002/L and Eosin, BioOptica #05-10002/L).

For IHC, slices were immuno-stained with polyclonal rabbit anti-Iba-1 antibody (Wako, #019-19741) and polyclonal rabbit anti-GFAP antibody (Novus Biologicals, #NB300-141). Any endogenous peroxidase activity was quenched by incubating the slices with 0.3% H_2_O_2_ in methanol for 10 min at RT. Both antibodies were used at a dilution of 1:1,000, incubated for 1 h at RT with EXPOSE rabbit specific HRP/DAB detection IHC kit (Abcam, #ab80437). DAB chromogen was than applied to each section for 5 min at RT and counterstained with Mayer’s hematoxylin, dehydrated and mounted with Eukitt (BioOptica, #09-00100). Positive and negative controls were run simultaneously. Slides were acquired with Aperio AT2 digital scanner at a magnification of 40× (Leica Biosystems). Region of interest (ROI) were drowned manually on half hemisphere for cortex, on the entire hippocampus, and on a selected region of the cerebellum. Percentage of positive cells were calculated using Aperio eSlide Manager (Leica Biosystems). From the data obtained (weak, medium and strong positive intensity), weak positive were not included in the analysis as considered as background.

### Statistical Analysis

Statistical evaluation of [^18^F]-VC701 tracer uptake was carried out using two-way ANOVA for Multiple Comparisons with a Tukey *post hoc* correction with gender, aging and treatment as covariates. Data representing radio ligand uptake in each LPS-treated and vehicle group were analyzed through parametric unpaired Student’s *t*-test. For gene expression studies, data were normalized to housekeeping gene followed by a fold change evaluation comparing data to internal control group. Data derived from comparison of LPS-treated groups were statistically analyzed using two-way ANOVA for Multiple Comparisons with a Tukey *post hoc* correction, while for those reported on Supplementary Figures, data were statistically evaluated using parametric unpaired Student’s *t*-test. Immunohistochemical analyses were performed using two-way ANOVA for Multiple Comparison with Tukey *post hoc* correction.

Analyses were performed using the Prism V6.0 software (GraphPad Prism, San Diego, CA, USA). Statistical significance was accepted when *p < 0.05, **p < 0.01, and ***p < 0.001.

## Results

### Peripheral Exposure to LPS Influences Sex- and Age-Dependent Microglia Activation

**Figure 2:**
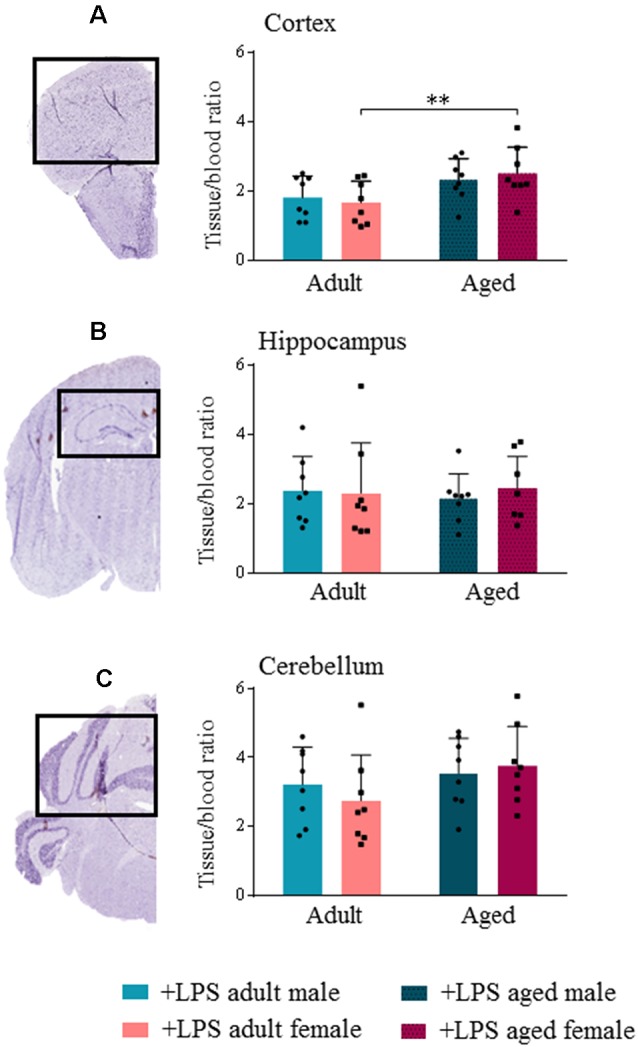
Translocator protein (TSPO) tracer uptake reveal microglial activation increased in aged female lipopolysaccharide (LPS)-treated animals. [^18^F]-VC701 brain tracer uptake in cortex **(A)**; hippocampus **(B)** and cerebellum **(C)** on LPS-treated adult and aged male and female mice. Data were analyzed using two-way ANOVA analysis with Tukey *post hoc* and are expressed as percentage (%) of injected dose per gram of tissue to blood ratio, mean ± SD, eight mice per group; Mouse brain stained slices (cortex, hippocampus and cerebellum) derived from immunohistochemistry (IHC) studies. *p < 0.05; **p < 0.01; ***p < 0.001.

TSPO expression in different brain areas of adult and aged untreated and LPS-treated male and female mice was evaluated by *ex vivo* binding of [^18^F]-VC701 to TSPO, 6 h after LPS/vehicle injection. LPS treatment induced a statistically significant increase (*p* < 0.05) of tracer’s uptake in the cortex and cerebellum of aged males, and in cortex and hippocampus of aged females (Supplementary Figure S1). Higher uptake of [^18^F]-VC701 was observed in the cortex of aged vs. adult LPS-treated females (Figure 2). This age-dependent effect was not observed in males. The percentage of tracer’s uptake in the cortex and hippocampal areas was significantly higher in LPS-treated aged females compared to age-matched vehicle (cortex 51.6%, *p* ≤ 0.017; hippocampus 86.4% *p* ≤ 0.010; cerebellum ns, 36.1%, data not shown). Tissue to blood ratio of tracer’s uptake in LPS-treated females, expressed as % of injected dose per gram of tissue (%I.D./g), is shown in Table 3.

**Table 3:** Tissue to blood [^18^F]-VC701 uptake ratio.

Brain Region	Tissue/blood ratio (%I.D./g; mean ± SD)	
	LPS-treated adult females	LPS-treated aged females
Cortex	1.6 ± 0.6	2.52 ± 0.7
Hippocampus	2.31 ± 1.4	2.46 ± 0.9
Cerebellum	2.75 ± 1.3	3.75 ± 1.1

### Female Brain Shows a Higher Pro-inflammatory Response to LPS During Aging

In order to confirm that the higher [^18^F]-VC701 uptake was associated with a pro-inflammatory response of microglia, we measured the levels of IL-1β, TNF-α, IL-6, IL-4 and Arg-1 transcripts in LPS-treated and untreated mice. In all brain regions, peripheral LPS injection induced neuroinflammation irrespective of sex and age, as shown by significantly increased transcript levels of IL-1β, IL-6 and TNF-α (Supplementary Figures S2A–C, S3A–C). Arg-1 and IL-4 transcripts were negligibly expressed in both LPS-treated and untreated mice (data not shown).

**Figure 3:**
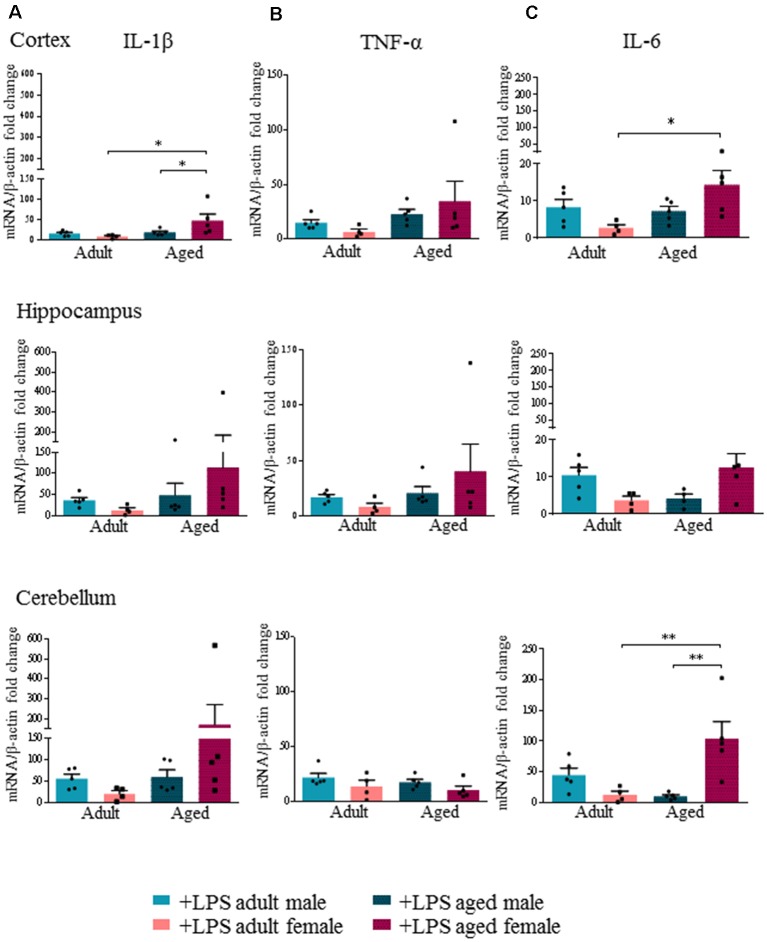
Increased expression levels of pro-inflammatory cytokines in aged LPS treated mice. Gene expression analysis of IL-1β **(A)**, TNF-α **(B)** and IL-6 **(C)** by RT-qPCR in cortex, hippocampus and cerebellum of all LPS-treated animals. All the values were normalized to mouse β-actin and expressed as fold change referred to internal control group. Bars represent the average of triplicate measurements and error bars represent ± SEM, five animals per group. Statistical analysis was performed using two-way ANOVA following by Tukey *post hoc* test of the relative mRNA expression of LPS-treated mice previously normalized to internal control group. *p < 0.05; **p < 0.01; ***p < 0.001.

In agreement with [^18^F]-VC701 uptake results, in female but not in male brain, age affected levels of the pro-inflammatory cytokines IL-1β, TNF-α and IL-6 in response to the LPS challenge. Aged females compared to adult females showed increased levels of IL-1β and IL-6 in all areas, which was statistically significant in the cortex for IL-1β, and in both cortex and cerebellum for IL-6 (Figures 3A,C). No age-dependent effects on IL-1β, TNF-α and IL-6 transcript levels were detected in males. Increased IL-1β transcript levels in the cortex and IL-6 in the cerebellum were detected in aged females compared to aged males (Figures 3A,C). TNF-α transcript levels were increased in the cortex and hippocampus of aged compared to adult females, although the differences were not statistically significant (Figure 3B).

### Sex and Age Effect on Transcript Levels of Markers of Microglia Activation

**Figure 4:**
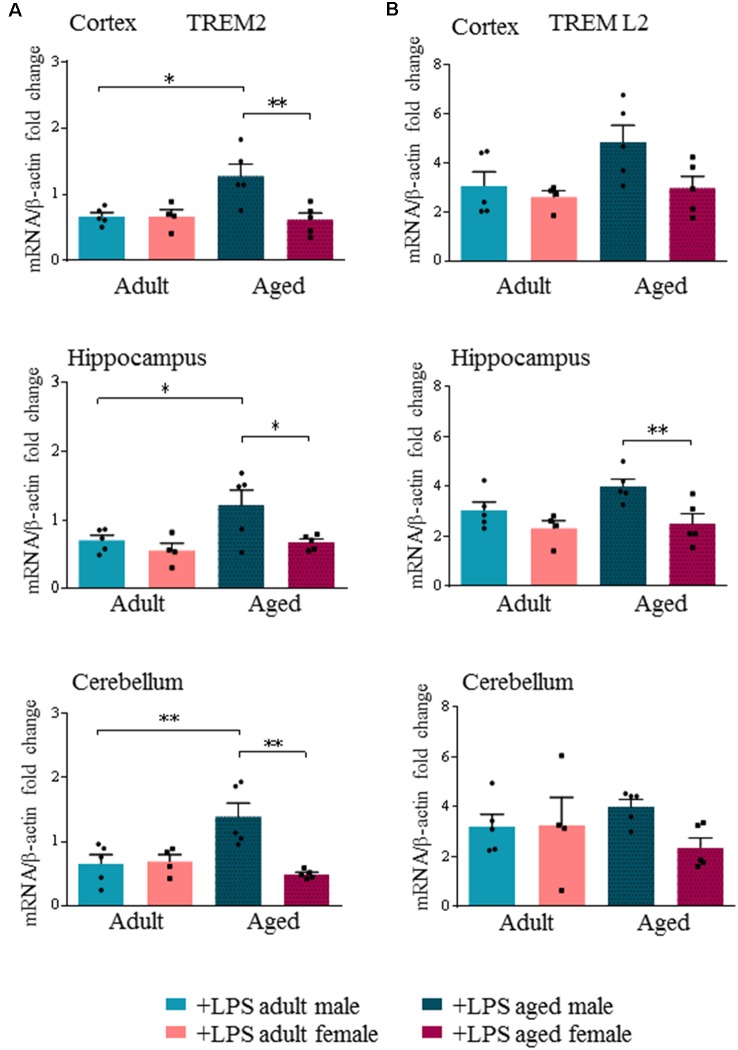
Triggering receptor expressed on myeloid cells like 2 (TREM2) is differentially expressed in aged LPS treated mice. Gene expression analysis of TREM2 **(A)**, TREML2 **(B)**, two immunomodulatory markers by RT-qPCR in cortex, hippocampus and cerebellum of all LPS-treated animals. All the values were normalized to mouse β-actin and expressed as fold change referred to internal control group. Bars represent the average of triplicate measurements and error bars represent ± SEM, five animals per group. Statistical analysis was performed using two-way ANOVA following by Tukey *post hoc* test of the relative mRNA expression of LPS-treated mice previously normalized to internal control group. *p < 0.05; **p < 0.01; ***p < 0.001.

Interestingly, we found an age- and sex-dependent effect on the expression of the neuro-inflammatory markers TREM2 and TREML2 after LPS administration. Our results showed that peripheral LPS injection induced a significant decrease of TREM2 transcript levels in all brain regions of adult males and females (Supplementary Figure S2D). Yet in aged males, LPS injection did not decrease TREM2 transcript levels, which were instead significantly decreased in all brain regions of aged females (Supplementary Figure S3D). When we specifically compared TREM2 transcript levels among LPS-treated mice, we found that they were significantly higher in aged males compared to aged females (*p* < 0.0002), while no differences were detected in adult mice of both sexes (Figure 4A).

TREML2 transcript levels were significantly increased in the hippocampus of aged males compared to aged females (*p* < 0.01), and also in the cortex, although not reaching statistical significance (Figure 4B, Supplementary Figures S2E, S3E).

### Microglia Activation and Astrogliosis in Aged Female Animals

**Figure 5:**
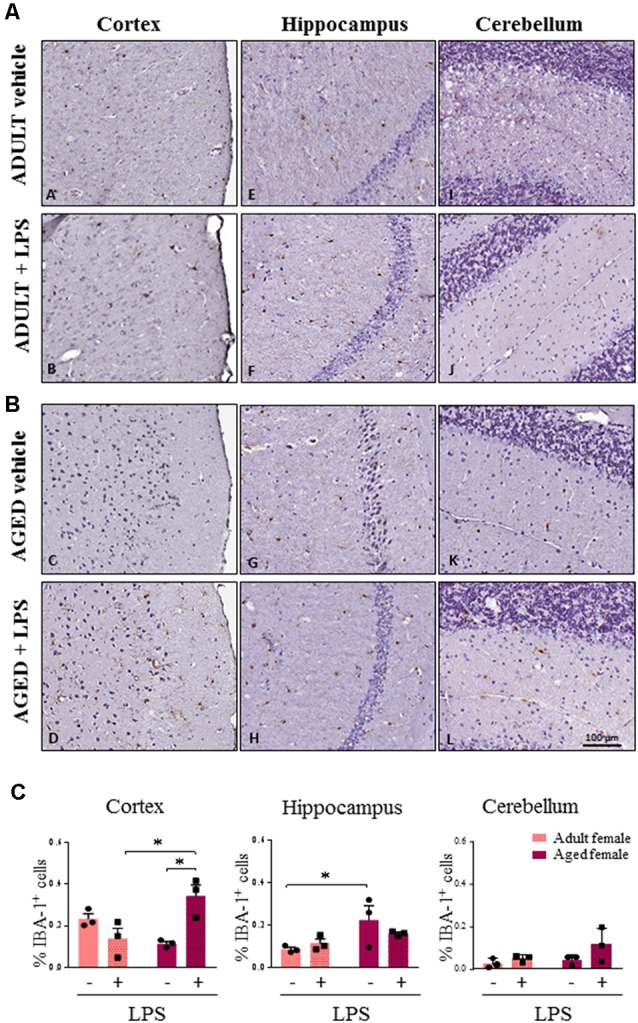
Increased IBA1 immunoreactivity and changes in glia morphology in brain areas of LPS-treated aged vs. LPS-treated adult females. **(A)** Immunostaining of Iba-1^+^ cells in cerebral cortex, hippocampus and cerebellum from one representative vehicle and one representative LPS-treated adult female mouse. **(B)** Immunostaining of Iba-1^+^ cells in cerebral cortex, hippocampus and cerebellum from one representative vehicle and one representative LPS-treated aged female mouse. **(C)** Quantification of the percentage of Iba-1^+^ cells in cortex, hippocampus and cerebellum of all female groups used in the study. Data are expressed as mean ± SEM of selected region of interest (ROI) performed on three animals per group, scale bar = 100 μm. *p < 0.05; **p < 0.01; ***p < 0.001.

As major differences in the neuro-inflammatory response to LPS were observed in females, we further evaluated glia activation in females by IHC. In all LPS-treated females, H&E staining showed no sign of neuronal loss or morphological changes in the three brain areas examined (cortex, cerebellum and hippocampus, data not shown). In LPS-treated aged females an increased percentage of Iba-1 positive cells were detected when compared to LPS-treated adult females, and vehicle-treated adult and aged females, which is indicative of a higher inflammatory status (Figure 5A, cortex). Microglia activation was suggested by cell morphology in the cerebral and cerebellar cortex of LPS-treated aged vs. LPS-treated adult females and vehicle-treated females (Figures 5A–C, cortex and cerebellum). In the hippocampus both LPS and vehicle-treated aged females showed a higher degree of Iba-1 immunoreactivity in comparison with adult females reaching statistical significance in vehicle-treated aged vs. vehicle-treated adult mice (Figure 5C, hippocampus).

**Figure 6:**
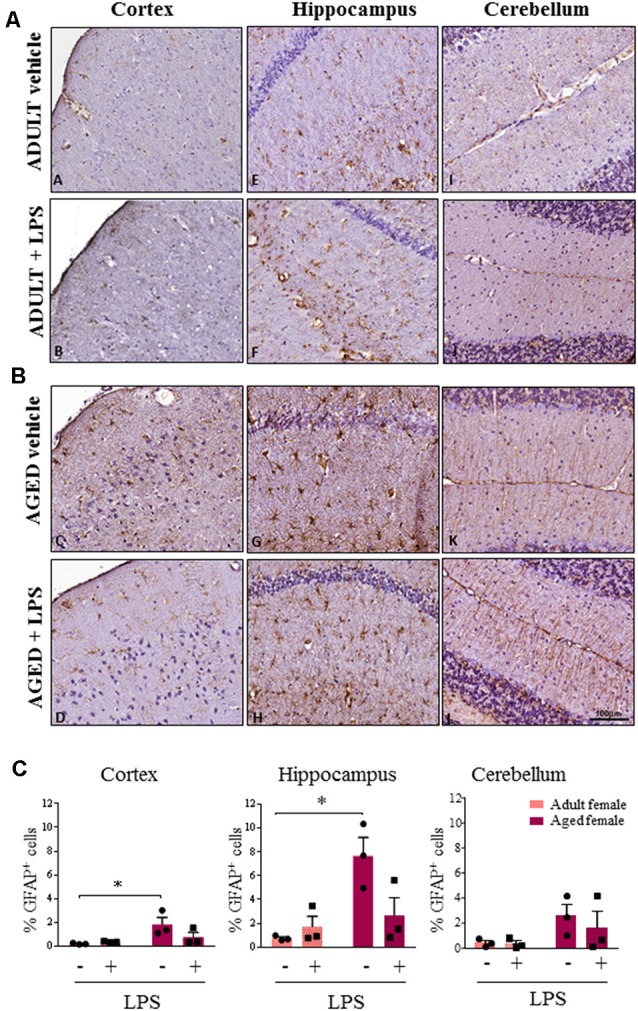
Increased astrocytosis and changes in glia morphology in brain areas of LPS-treated and vehicle-treated aged females. **(A)** Immunostaining of GFAP^+^ cell in cerebral cortex, hippocampus and cerebellum from one representative vehicle and one representative LPS-treated adult female mouse. **(B)** Immunostaining of GFAP^+^ astrocyte cells in cerebral cortex, hippocampus and cerebellum from one representative vehicle and one representative LPS-treated aged female mouse. **(C)** Quantification of percentage of GFAP^+^ cells in cortex, hippocampus and cerebellum of all female groups used in the study. Data are expressed as mean ± SEM of selected ROI performed on three animals per group, scale bar = 100 μm. *p < 0.05; ** *p* < 0.01; ***p < 0.001.

Astroglial cells showed an increase in GFAP immunoreactivity in aged compared to adult females (Figures 6A,B). This observation was consistent in the cortex, cerebellum and hippocampus of aged females where astrocytes also showed hyperplasia of cytoplasm and of cellular processes resembling an activated phenotype. Quantification analysis showed a significant higher percentage of GFAP positive cells in cortex and hippocampus of aged compared to adult females. A similar trend was observed also in the cerebellar region (Figure 6C). Furthermore, in the sections where olfactory bulb was present, a general increase in immunoreactivity for both Iba-1 and GFAP was observed, again more evident in LPS-treated aged females for Iba-1 and in vehicle and LPS-treated aged females for GFAP (data not shown).

## Discussion

In this study, we demonstrate for the first time that both age and sex influence an early neuro-inflammatory response after an acute mild peripheral LPS challenge in mice. In comparison to control conditions, LPS-treated aged male and female mice showed an increase [^18^F]-VC701 binding indicative of higher expression of TSPO. A prominent effect was observed in LPS-treated aged compared to adult females. [^18^F]-VC701 is a structural analog of PK11195, which targets 18 kDa TSPO (Di Grigoli et al., 2015). TSPO’s main role is to transport cholesterol across the outer mitochondrial membrane. While TSPO is negligibly expressed in the normal brain parenchyma, its expression increases upon microglia activation and macrophage infiltration (Betlazar et al., 2018). Thus TSPO up-regulation has been mainly associated with a pro-inflammatory status of microglia (Bonsack et al., 2016). PET-TSPO has been successfully applied to image the neuro-inflammatory reaction to LPS administration in the brain of human subjects, showing an increased binding of the TSPO radiotracer [^11^C]-PBR28 in all brain regions (Sandiego et al., 2015). Co-localization of TSPO with CD206-positive alternatively-activated microglia has also been reported (Liu et al., 2015). Our results, although obtained from analysis of whole tissue are in agreement with transcriptomic data of purified microglia (Villa et al., 2018). Villa et al. (2018) demonstrated that microglia cells were sexually differentiated in the adult brain, as male mice carried a phenotype more poised to inflammatory reactions than female microglia. Moreover, in females, microglia displayed neuroprotective features in an acute pre-clinical model of ischemic insult (Villa et al., 2018). Gene sexual dimorphism has also been reported in humans by Berchtold et al. (2008). This study shows that males display a greater number of gene expression changes (mainly downregulation) during the transition to the sixth and seventh decade of life while females show more prominent gene expression changes later in life, during the eighth and ninth decade of life. Moreover, increased immune activation was greater in the female brain, as shown by a higher percentage of up-regulated genes involved in the immune response and inflammation (34% in males and 50% in females; Berchtold et al., 2008).

To better understand the nature of the microglia response, we evaluated the effect of the peripheral LPS challenge on transcription of a few inflammatory mediators: IL-1β, TNF-α, IL-6, Arg1 and IL-4. We showed that LPS induced a pro-inflammatory reaction in the brain of male and female mice as indicated by the up-regulation of IL-1β, TNF-α and IL-6 gene expression. However, in agreement with [^18^F]-VC701 studies, the effect of LPS on pro-inflammatory cytokine transcript levels was significantly increased in aged compared to adult females, as well as in aged females compared to aged males.

As a major age effect was observed in females, we further analyzed microglia and astrocyte status by IHC in all brain regions of adult and aged females. In agreement with TSPO expression and pro-inflammatory cytokine transcript levels, we found an increased expression of microglia and astrocyte markers in LPS-treated and vehicle-treated aged compared to adult females, with some regional differences. Increased Iba-1 expression was present particularly in the cerebral cortex of LPS-treated aged animals, whereas GFAP immunoreactivity was higher in aged females in all the cerebral areas examined, regardless of LPS administration. Finally, the higher Iba-1 and GFAP signals detected in aged females was associated with a different cellular morphology. Altogether, our results highlight the acute onset of a higher neuro-inflammatory response in aged females. A similar response was also reported in female 3xTg-AD mice, where neuroinflammation was associated with progressive cognitive decline that increased as a function of age (Belfiore et al., 2019).

Microglia, as the primary source of pro-inflammatory cytokines, are pivotal mediators of neuroinflammation (Colonna and Butovsky, 2017). The amplitude and duration of the context-dependent activation of microglia are regulated by many pattern-recognition receptors, and immune receptors that deliver either activating or inhibitory signals. TREM2 and TREML2 are two microglia/monocyte regulators with potential and opposite roles in neurodegenerative disorders such as AD (Zsido et al., 2017). We show that acute peripheral administration of LPS induced an opposite modulation of TREM2 and TREML2 transcripts. In all groups analyzed, except aged male mice, we showed that LPS reduced TREM2 while increasing TREML2 transcript levels. TREM2 regulates critical functions of microglia including inhibition of pro-inflammatory responses and stimulation of phagocytosis of apoptotic neurons (Piccio et al., 2008). TREM2-transduced myeloid precursors mediate nervous tissue debris clearance and facilitate recovery in an animal model of multiple sclerosis (Takahashi et al., 2005, 2007). Moreover, TREM2 binds poly-anionic ligands including LPS (Wang et al., 2015). Previous findings showed that LPS stimulation suppressed TREM2 levels in microglia, at the same time inducing pro-inflammatory cytokines secretion (Zheng et al., 2016). Peripheral LPS challenge (at a similar dose to that used in our study) induced up-regulation of TREML2 and down-regulation of TREM2 levels in microglia, and increase of pro-inflammatory cytokines (Zheng et al., 2016; Zhang et al., 2017). In particular, reduction of TREM2 was associated with a decreased microglia proliferation, an effect that was absent when TREML2 was knocked down.

During aging, microglia undergo cellular senescence losing regenerative and trophic functions (Mecca et al., 2018). According to Colonna and Wang (2016) TREM2 protects the aged brain from insults. Indeed, carrying a loss of function mutations in the TREM2 gene represents an important risk factor for dementia (Gao et al., 2017). *In vitro* experiments demonstrated that, silencing of TREM2 exacerbated LPS-induced pro-inflammatory response in BV2 microglia (Zhong et al., 2015), In contrast, overexpression of TREM2 lowered the pro-inflammatory response, thus supporting the hypothesis that TREM2 expression modulates the acute and transient pro-inflammatory responses induced by LPS (Zhong et al., 2015). Thus, the higher TREM2 expression observed in aged male mice might represent a strategy to counterbalance the pro-inflammatory milieu of aging, partially sustained by activation of signaling pathways mediated by TREML2, which likely activates apoptosis and modulates microglial functions (de Freitas et al., 2012; Zheng et al., 2016).

In aged female brains, we observed up-regulation of reactive GFAP-positive astroglia in all the regions examined independently from LPS administration. Although GFAP expression is limited to only a fraction of astrocytes with a substantial regional heterogeneity, its expression has been associated with reactive astrogliosis (Verkhratsky and Nedergaard, 2018). During aging, high levels of GFAP expression were identified as “mild to moderate astrogliosis” while the presence of cellular hypertrophy in addition to GFAP increase appears to be associated to “severe diffuse astrogliosis” (Cohen and Torres, 2019). Matias et al. (2019) reported that the astrocyte response to brain aging showed intra-regional heterogeneity (Matias et al., 2019): astrocytes in the hippocampal region presented an age-dependent hypertrophy, similar to the astrocyte morphology detected in our study, which may contribute to synapse loss and neuroinflammation.

Interestingly, increased levels of neuro-inflammatory markers, including GFAP, are present in the brain of AD patients. Recently Barroeta-Espar et al. (2019) demonstrated reduced levels of GFAP and a different pattern of cytokines expression in subjects resilient to AD pathology. This selected population of aged subjects harbor plaque and tangle loads that in some cases are equivalent to those found in AD, although in absence of the typical patterns of neuronal/synaptic loss seen in the pathology. These findings suggest that down-regulation of neuroinflammation is one of the differential traits of human brain resilience to AD pathology, a mechanism that might be less efficient in female brain. Implications of our findings may impact the development of clinical phenotypes such as delirium and dementia. Delirium is one of the most common neuropsychiatric conditions among older people, which is known to develop as an acute consequence of peripheral infection or inflammation originating outside the brain (Cerejeira et al., 2010; Franceschi and Campisi, 2014; Bu et al., 2015; Morandi et al., 2018). The higher the level of frailty, the higher the likelihood of developing delirium in reaction to an inflammatory stimulus (Bellelli et al., 2017; Persico et al., 2018). Neuroinflammation has also been recognized as a pathophysiological mechanism of dementia, especially AD, again with frailty as a modulating factor of the disease clinical expression (Tao et al., 2018; Wallace et al., 2019).

Future studies should explore chronic neuroinflammation and morphological-functional changes in glial cell upon repetitive systemic insult or an acute severe neural event. As the cortex represents in our study the most important region influenced by sex and age, we will further explore gene modulation of specific pathways trough transcriptomic analysis of cortical glia in adult and aged female and male mice. Furthermore, specific behavioral phenotypes such as attention and active inhibitory processes associated with cognitive frailty will be investigated.

Finally, it would be appropriate to ascertain if age- and sex-specific patterns of neuroinflammation could be identifiable in humans too, and to clarify the mechanisms involved in such differences.

## Conclusion

In conclusion, the results of this study show that the neuro-inflammatory response of a mouse’s brain to an acute peripheral LPS challenge is sex- and age-dependent, and is likely to involve multiple cellular and molecular regulators of neuronal functions. Our results might set the basis for further studies aimed at characterizing sex-related targets involved in the modulation of the neuro-inflammatory response in aging. This knowledge could be relevant for the treatment of conditions such as delirium and dementia.

## Data Availability Statement

The raw data supporting the conclusions of this manuscript will be made available by the authors, without undue reservation, to any qualified researcher.

## Ethics Statement

The animal study was approved by our Institutional Guidelines for the Care and Use of Experimental Animals (IACUC) and the National Law for animals used in research (Prot. N. SK552/2012 D.lsg. 116/1992 and N. 722/2016-PR D.lsg. 26/2016).

## Author Contributions

VMu: data generation, collection, analysis and interpretation, and manuscript drafting. SB and GG: data generation and collection. MP: support in qRT-PCR data generation and analysis. EB, VR-M, and PM: support in IHC data analysis and interpretation. AC, VMa, and CM: production and validation of the tracer [^18^F]-VC701. GB: critical review of the manuscript. PP-B and RM: study conception and design, manuscript preparation. All the authors approved the final version of the manuscript.

## Conflict of Interest

The authors declare that the research was conducted in the absence of any commercial or financial relationships that could be construed as a potential conflict of interest.
