# Supplementary material for: Age and Sex Influence the Neuro-inflammatory Response to a Peripheral Acute LPS Challenge
Source: Front Aging Neurosci. 2019 Nov 5;11:299. doi: 10.3389/fnagi.2019.00299 (PMC6848890; doi:10.3389/fnagi.2019.00299)
Supplement: Supplementary file 1 [file Presentation_1.PPTX]

## Slide 1
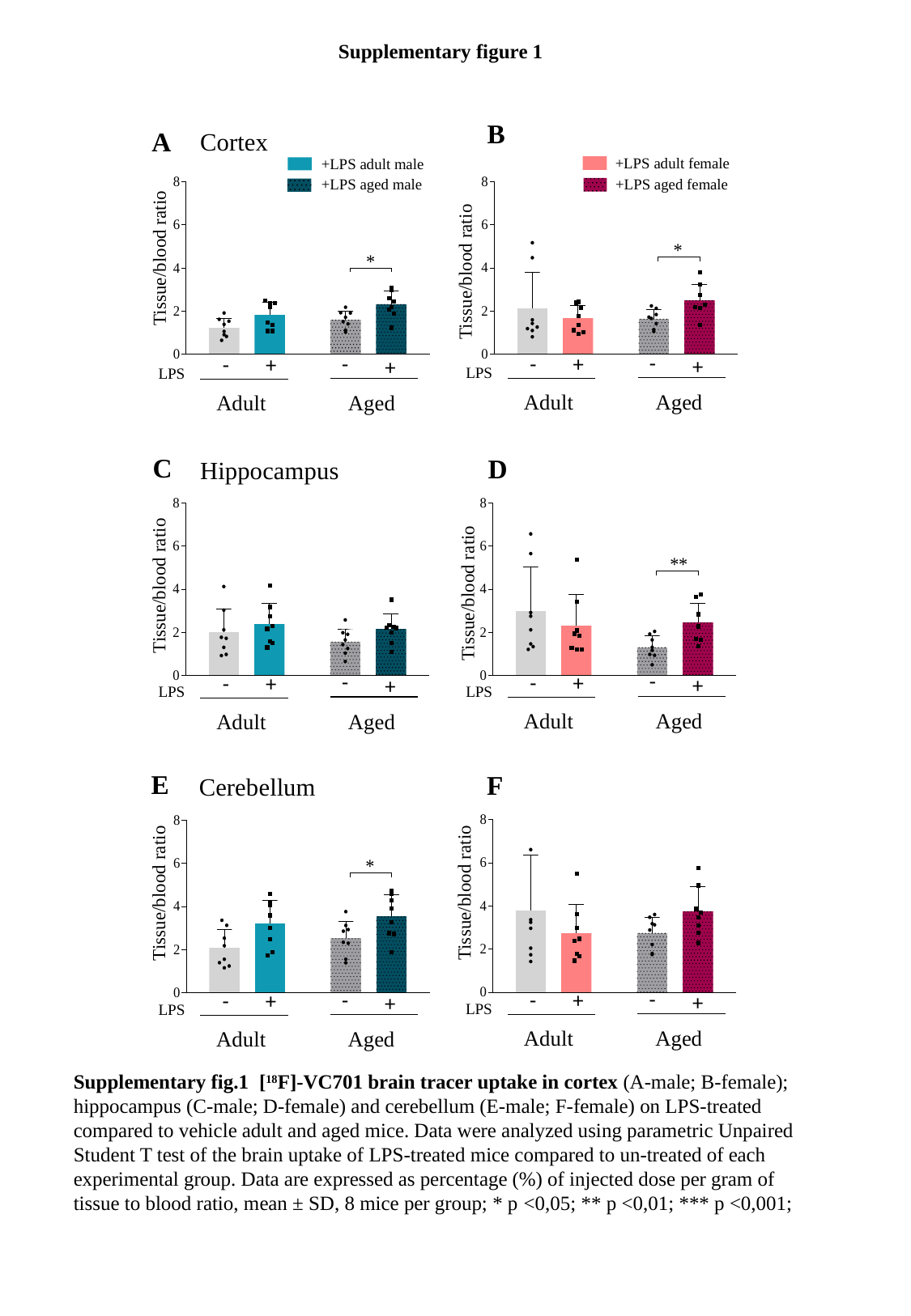

Supplementary figure 1
B
A
Cortex
Tissue/blood ratio
Tissue/blood ratio
-
-
+
+
LPS
Adult
Aged
-
-
+
+
LPS
Adult
Aged
C
D
Hippocampus
Tissue/blood ratio
Tissue/blood ratio
-
-
+
+
LPS
Adult
Aged
-
-
+
+
LPS
Adult
Aged
E
F
Cerebellum
Tissue/blood ratio
Tissue/blood ratio
-
-
+
+
LPS
Adult
Aged
-
-
+
+
LPS
Adult
Aged
+LPS adult female
+LPS adult male
+LPS aged male
+LPS aged female
Supplementary fig.1 [18F]-VC701 brain tracer uptake in cortex (A-male; B-female); hippocampus (C-male; D-female) and cerebellum (E-male; F-female) on LPS-treated compared to vehicle adult and aged mice. Data were analyzed using parametric Unpaired Student T test of the brain uptake of LPS-treated mice compared to un-treated of each experimental group. Data are expressed as percentage (%) of injected dose per gram of tissue to blood ratio, mean ± SD, 8 mice per group; * p <0,05; ** p <0,01; *** p <0,001;

## Slide 2
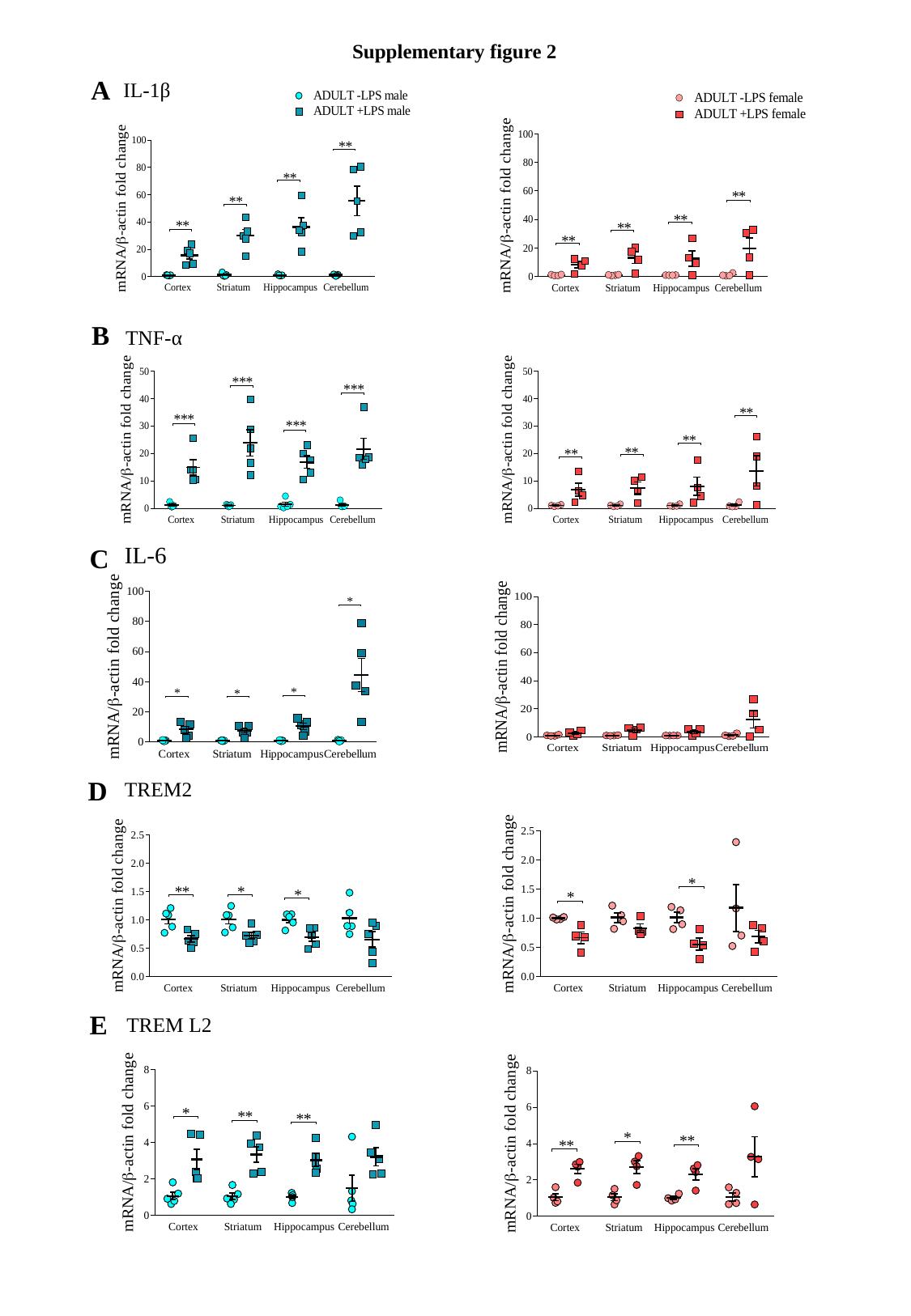

Supplementary figure 2
A
IL-1β
B
TNF-α
IL-6
D
TREM2
E
TREM L2
C

## Slide 3
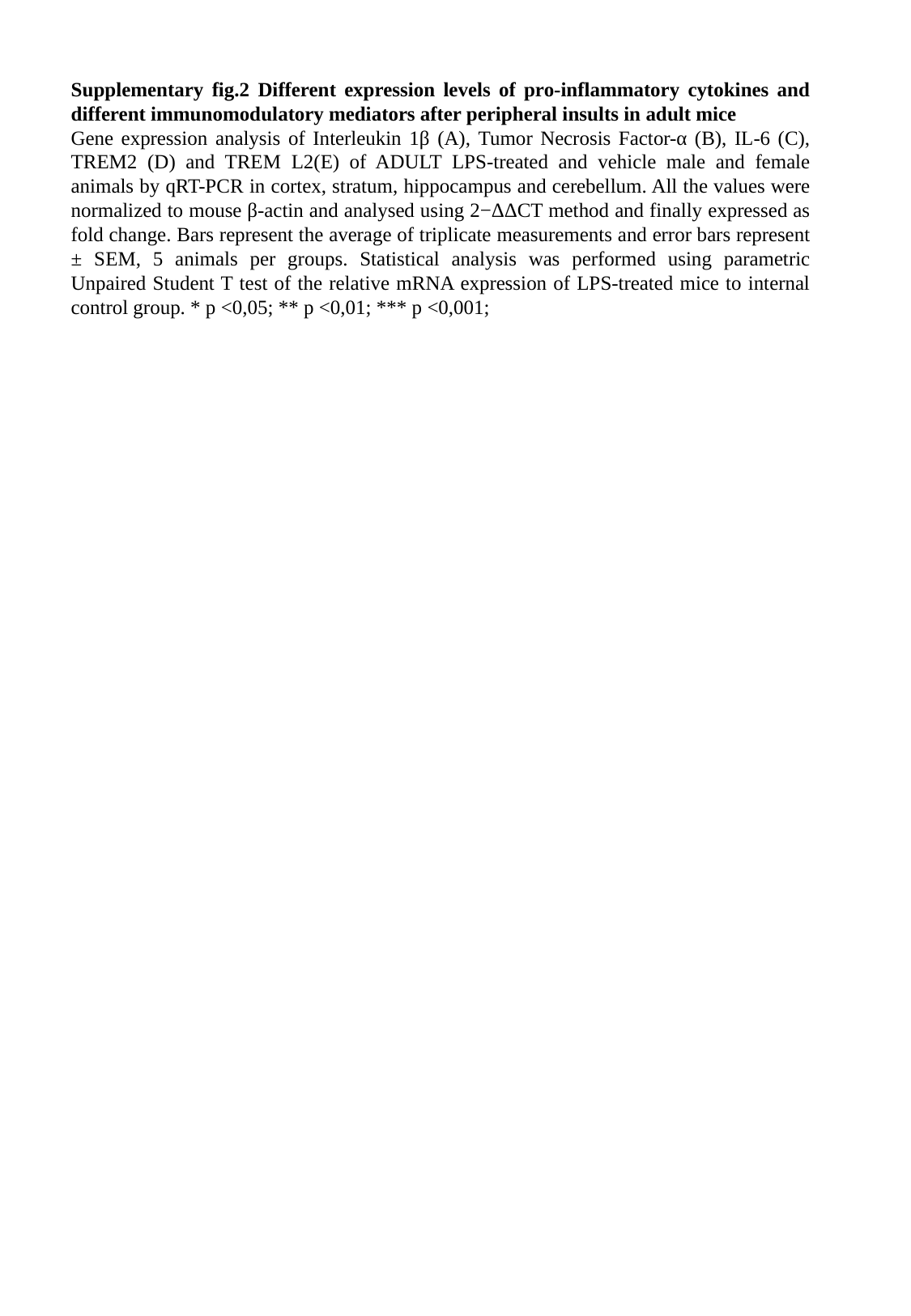

Supplementary fig.2 Different expression levels of pro-inflammatory cytokines and different immunomodulatory mediators after peripheral insults in adult mice
Gene expression analysis of Interleukin 1β (A), Tumor Necrosis Factor-α (B), IL-6 (C), TREM2 (D) and TREM L2(E) of ADULT LPS-treated and vehicle male and female animals by qRT-PCR in cortex, stratum, hippocampus and cerebellum. All the values were normalized to mouse β-actin and analysed using 2−ΔΔCT method and finally expressed as fold change. Bars represent the average of triplicate measurements and error bars represent ± SEM, 5 animals per groups. Statistical analysis was performed using parametric Unpaired Student T test of the relative mRNA expression of LPS-treated mice to internal control group. * p <0,05; ** p <0,01; *** p <0,001;

## Slide 4
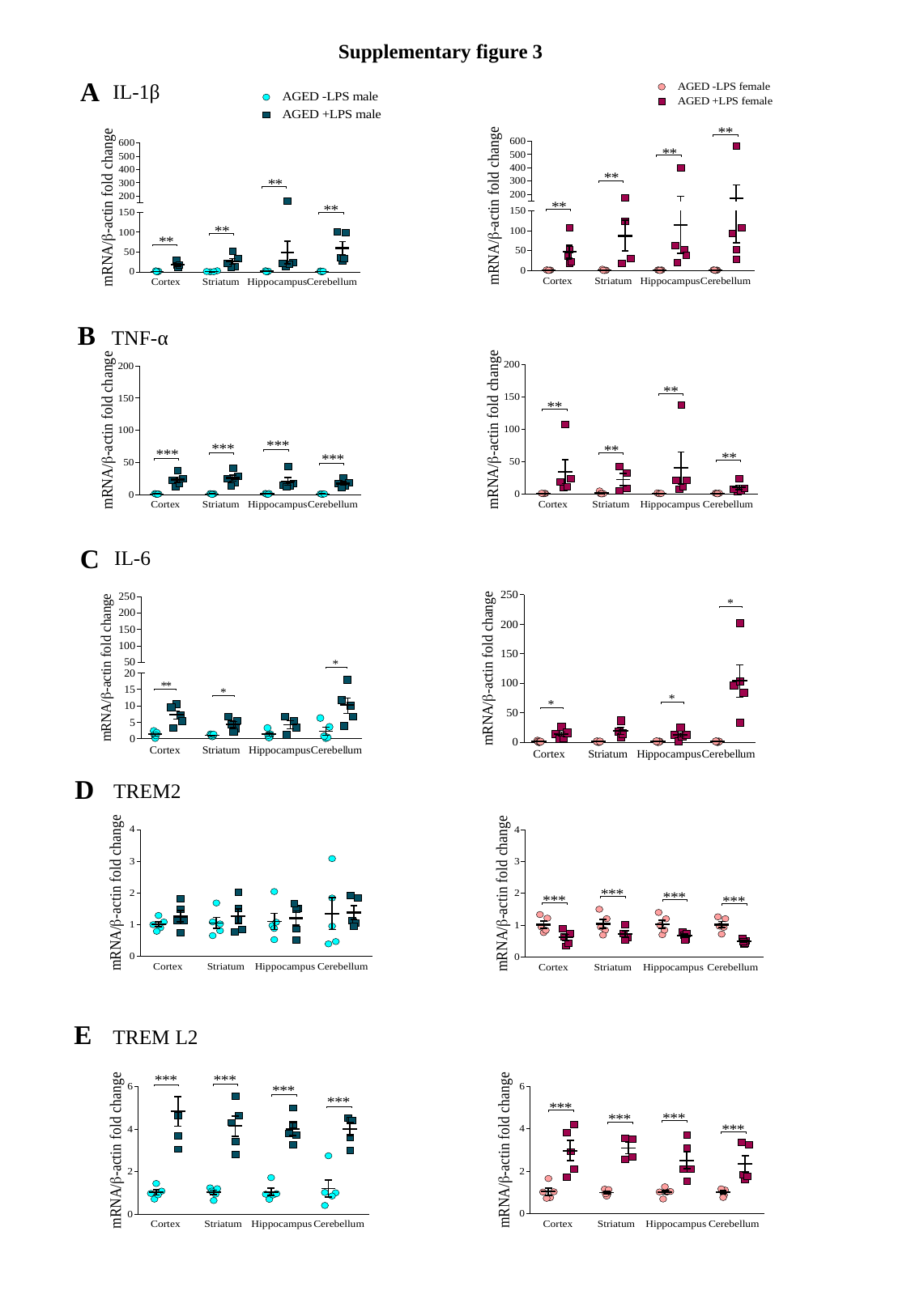

Supplementary figure 3
A
IL-1β
B
TNF-α
C
IL-6
D
TREM2
E
TREM L2

## Slide 5
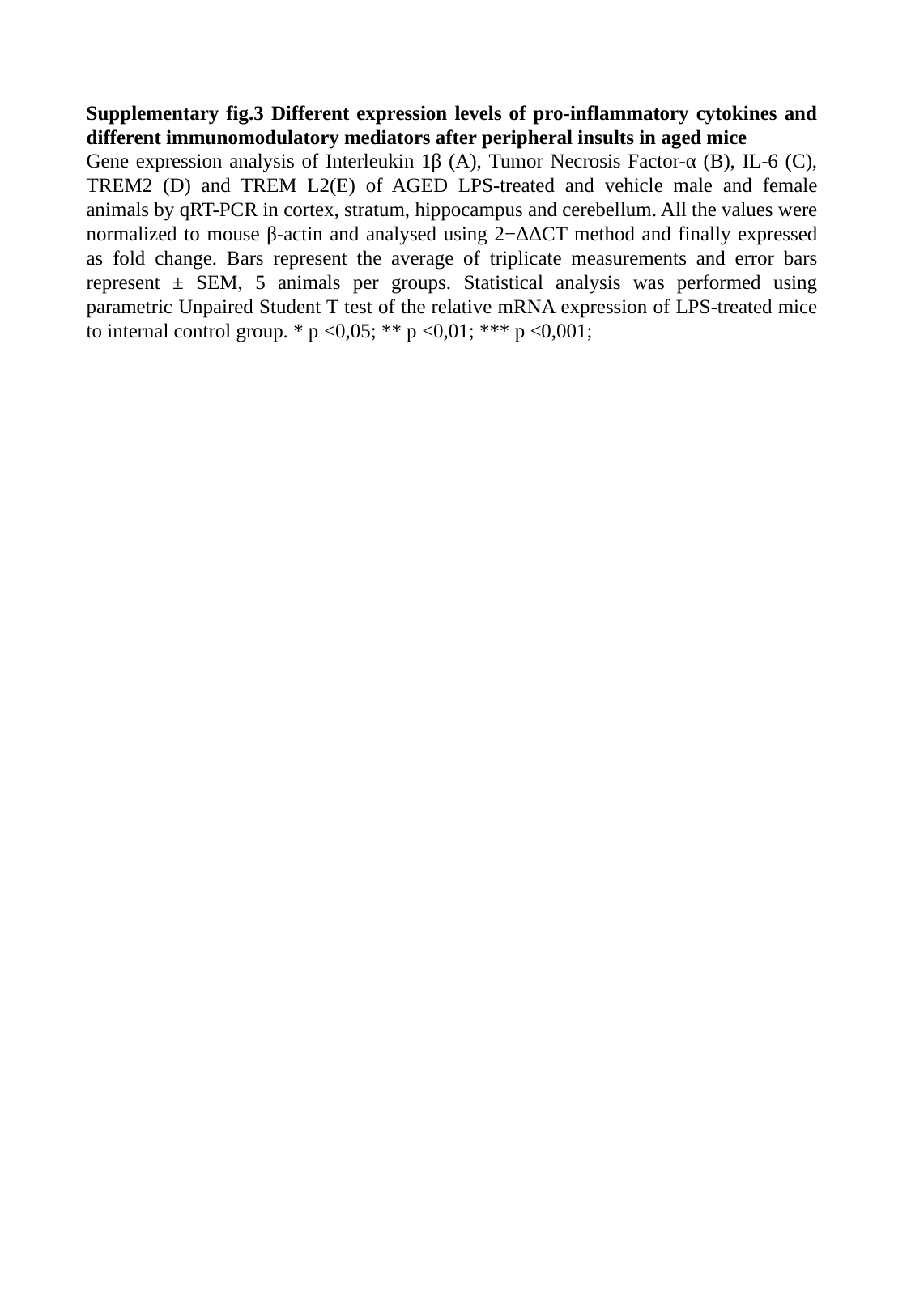

Supplementary fig.3 Different expression levels of pro-inflammatory cytokines and different immunomodulatory mediators after peripheral insults in aged mice
Gene expression analysis of Interleukin 1β (A), Tumor Necrosis Factor-α (B), IL-6 (C), TREM2 (D) and TREM L2(E) of AGED LPS-treated and vehicle male and female animals by qRT-PCR in cortex, stratum, hippocampus and cerebellum. All the values were normalized to mouse β-actin and analysed using 2−ΔΔCT method and finally expressed as fold change. Bars represent the average of triplicate measurements and error bars represent ± SEM, 5 animals per groups. Statistical analysis was performed using parametric Unpaired Student T test of the relative mRNA expression of LPS-treated mice to internal control group. * p <0,05; ** p <0,01; *** p <0,001;
